# Immunology and Donor-Specific Antibodies in Corneal Transplantation

**DOI:** 10.1007/s00005-021-00636-3

**Published:** 2021-11-06

**Authors:** Joanna Major, Bartosz Foroncewicz, Jacek Paweł Szaflik, Krzysztof Mucha

**Affiliations:** 1 Independent Public University Eye Hospital, Warsaw, Poland; 2 Department of Immunology, Transplantology and Internal Diseases, Medical University of Warsaw, Warsaw, Poland; 3 Department of Ophthalmology, Medical University of Warsaw, Warsaw, Poland; 4 Institute of Biochemistry and Biophysics, Polish Academy of Sciences, Warsaw, Poland

**Keywords:** Corneal transplantation, DSA, HLA, Keratoplasty, Rejection

## Abstract

The first human corneal transplantation was performed in 1905 by Eduard Zirm in the Olomouc Eye Clinic, now Czech Republic. However, despite great advancements in microsurgical eye procedures, penetrating keratoplasty in high-risk patients (e.g., vascularized or inflamed corneal tissue, consecutive transplants) remains a challenge. The difficulty is mainly due to the risk of irreversible allograft rejection, as an ocular immune privilege in these patients is abolished and graft rejection is the main cause of corneal graft failure. Therefore, tailored immunosuppressive treatment based on immunological monitoring [e.g., donor-specific antibodies (DSA)] is considered one of the best strategies to prevent rejection in transplant recipients. Although there is indirect evidence on the mechanisms underlying antibody-mediated rejection, the impact of DSA on cornea transplantation remains unknown. Determining the role of pre-existing and/or de novo DSA could advance our understanding of corneal graft rejection mechanisms. This may help stratify the immunological risk of rejection, ultimately leading to personalized treatment for this group of transplant recipients.

## Introduction

The cornea is the most frequently transplanted solid tissue. In 2020, the Eye Bank Association of America distributed 66,278 tissues for keratoplasty compared to 33,309 solid organ transplants (SOTs) performed in 2020 in the United States (Eye Bank Association of America: https://restoresight.org/what-we-do/publications/statistical-report/; U.S. Government Information on Organ Donation and Transplantation: https://optn.transplant.hrsa.gov/news/annual-record-trend-continues-for-deceased-organ-donation-deceased-donor-transplants). At the same time, 1269 keratoplasties were performed in Poland compared to 1180 SOTs (Poltransplant: https://www.poltransplant.org.pl/statystyka_2020.html).

**Fig. 1:**
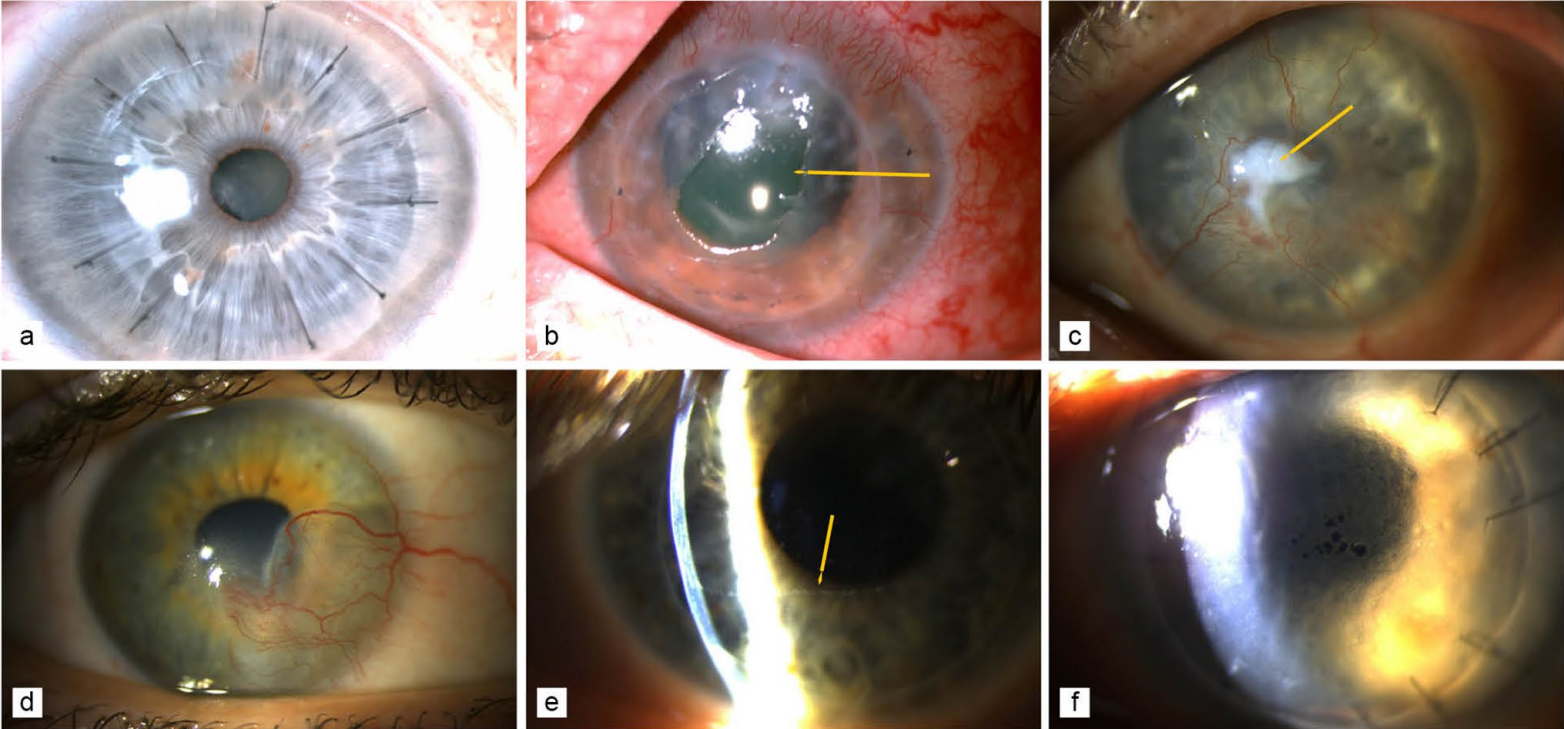
High-risk corneal recipients. **a** Translucent corneal graft (low-risk corneal recipient). **b** Loss of graft transparency with central erosion (arrow). **c** Neovascularization in corneal ulceration (arrow). **d** Scar with corneal neovascularization after herpes keratitis. **e** Corneal graft rejection precipitates (arrow) formed by white blood cells on the endothelium (Khodadoust line). **f** Corneal graft rejection with corneal edema and loss of graft translucency

**Fig. 2:**
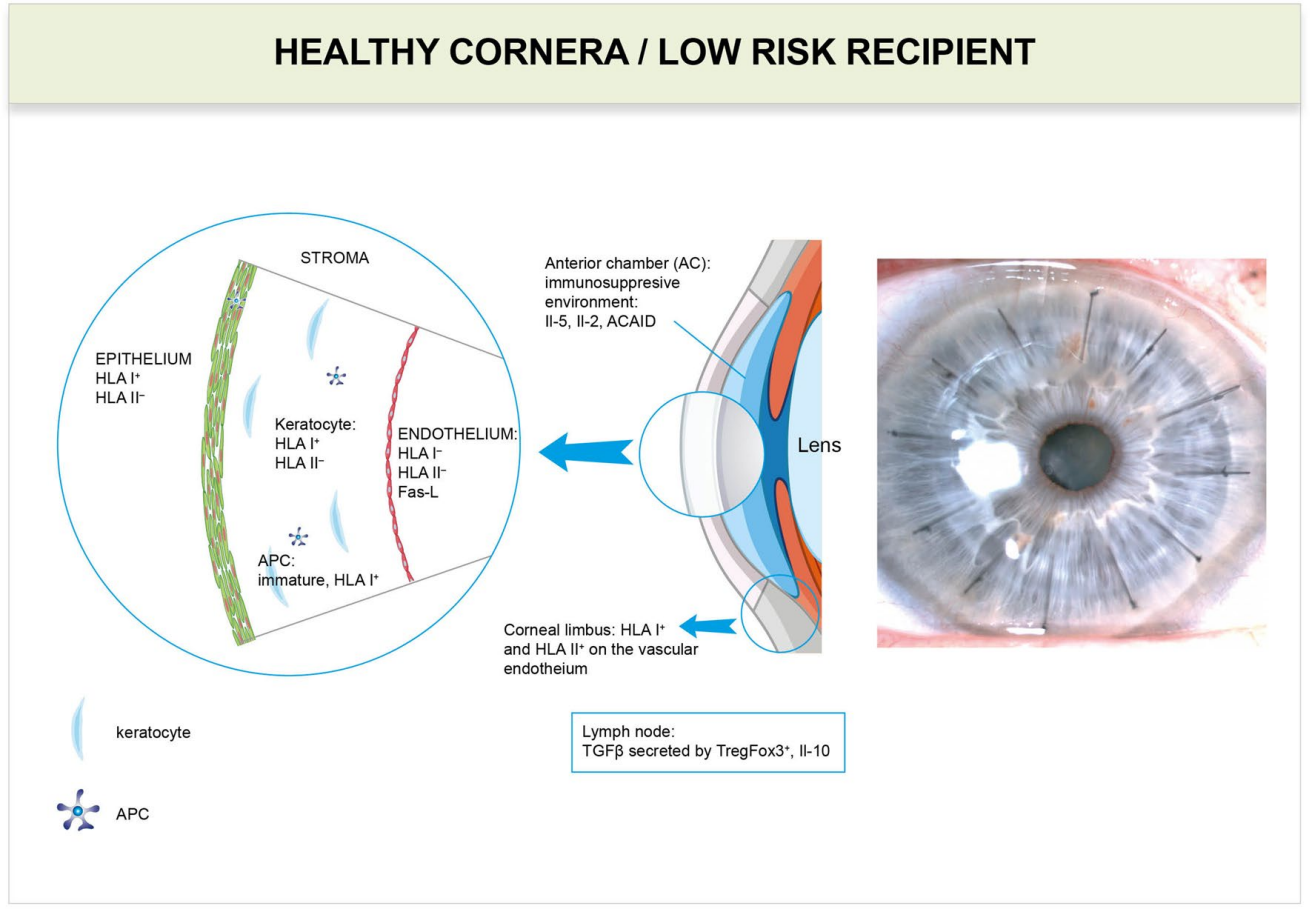
Low-risk corneal recipients/healthy cornea. In healthy corneas (and in grafts of low-risk recipients) only HLA I antigens are detected on corneal epithelial cells and keratocytes and are not detected on endothelial cells (neither HLA I nor HLA II). HLA I and HLA II are found only on the vascular endothelium in the corneal limbus (Whitsett and Stulting 1984). In the central part of the healthy cornea, there are no APCs or other inflammatory cells, as they could cause loss of its unique optic properties. There are only small numbers of immature APCs in the epithelium and in stroma near the limbus (Knickelbein et al. 2009; Kuffová et al. 1999). The immunosuppressive environment of the anterior chamber is based on the anterior chamber-associated immune deviation [ACAID], and IL-2 and IL-5 have graft protective effects (Maier et al. 2011). FasL is expressed on the corneal epithelial and endothelial cell and causes apoptosis of Fas^+^ limfoid cells (Stuart et al. 1997). In lymph nodes, draining the eye in the presence of IL-10 and transforming growth factor [TGFβ] secreted by T regulatory (Treg) cells expressing Foxp3 also has graft protective effects (Janyst et al. 2020)

**Fig. 3:**
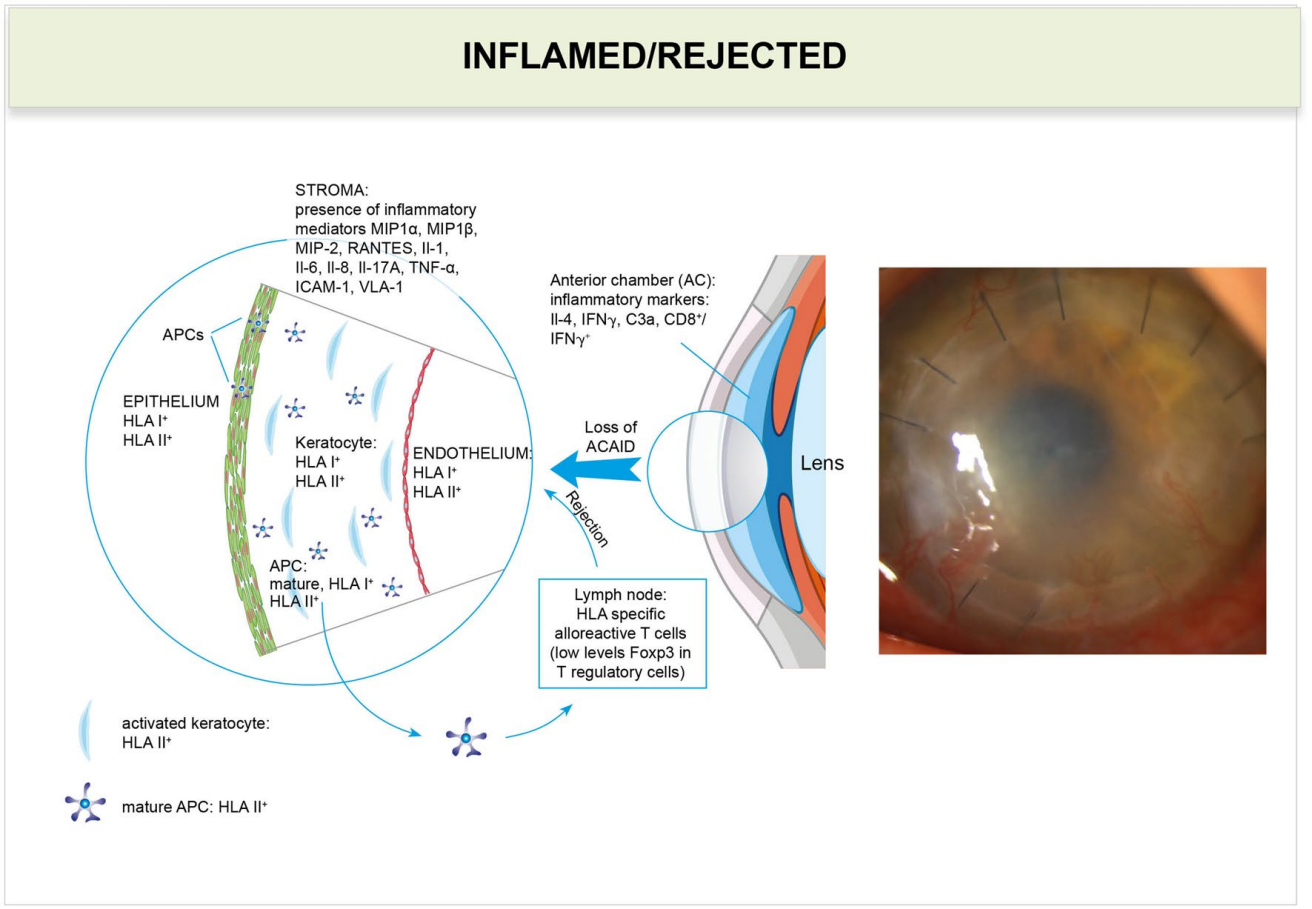
Inflamed/rejected corneal transplant. In inflamed or rejected corneas, HLA antigens are induced on endothelial cells. HLA II antigens are expressed on epithelial cells, stromal cells (keratocytes), and endothelial cells (Delbosc et al. 1990). In the stroma, there is a large number of activated keratocytes expressing HLA II and mature APCs expressing HLA II and lymphocytes (Th1) (Schönberg et al. 2020). In the aqueous humor of the anterior chamber, the balance between anti- and pro-inflammatory molecules is disrupted and hazardous factors are present such as IL-4, interferon γ (IFN γ), C3a, and CD8^+^/IFNγ^+^ (Maier et al. 2011; Yoon et al. 2019). The upregulation of inflammatory cytokines (IL-1, IL-6, IL-8, IL-17A, tumor necrosis factor [TNF-α]), pro-inflammatory chemokines (macrophage inflammatory protein 1 alpha [MIP-1α], MIP-1β; regulated on activation, normal T cell expressed and secreted [RANTES]), and adhesion molecules (intercellular adhesion molecule 1 [ICAM 1], very late antigen [VLA 1] attract APCs to the central part of the cornea and promote their maturation (expression of MHC II, CD80^+^, CD86^+^). Mature APCs (HLA I^+^, HLA II^+^) present donor antigens to naïve T cells in lymph nodes. After their clonal expansion, effector T cells (Th1CD4^+^/IFNγ) produce cytokines IL-2, IFN-γ, and TNF-α. These cells and cytokines lead to the apoptosis of endothelial cells. A small number of endothelial cells cause corneal edema and loss of graft translucency (Hong et al. 2001; Zhu et al. 1999; Zhu and Dana 1999)

**Fig. 4:**
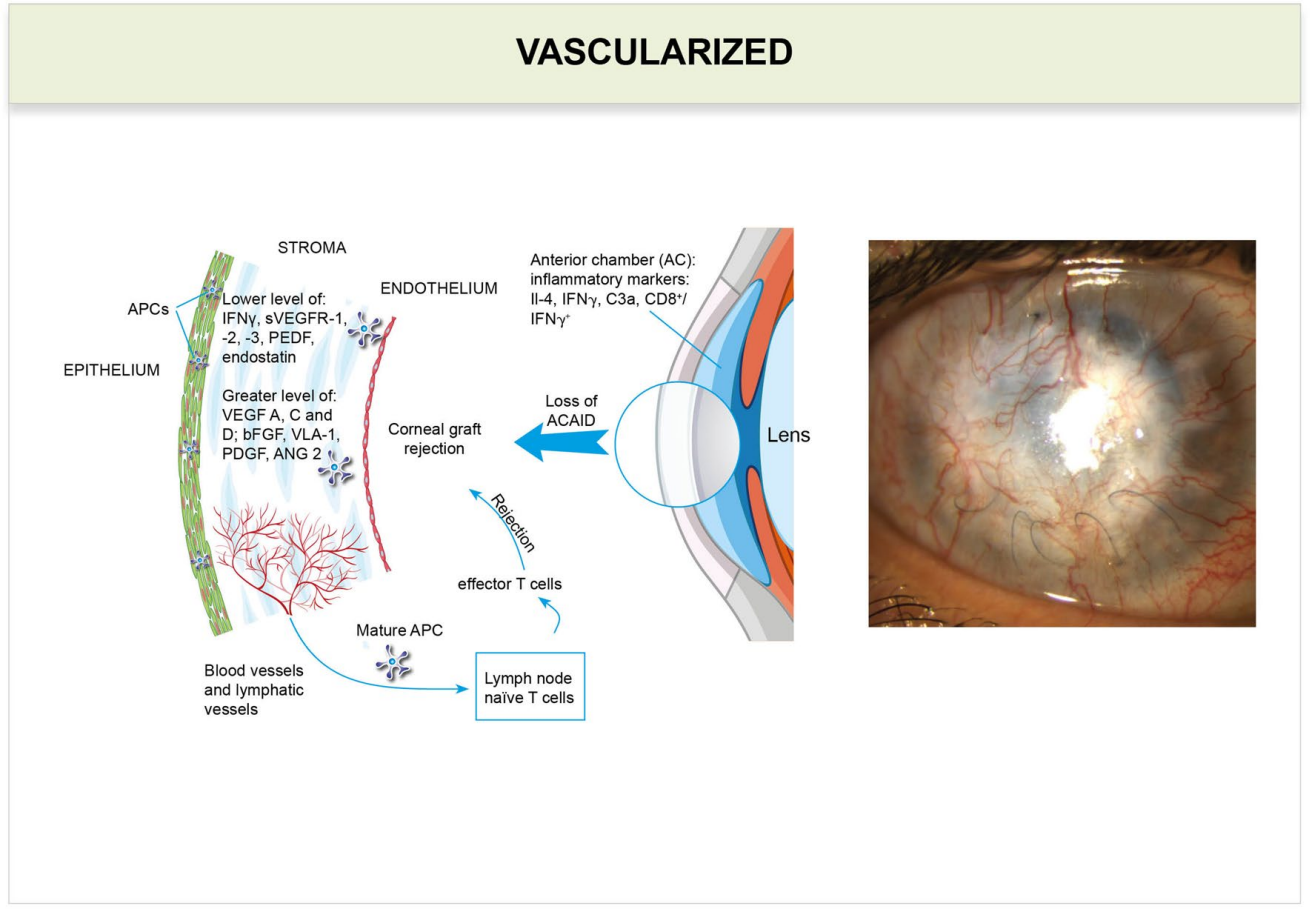
Vascularized corneal graft. Higher levels of proinflammatory mediators affect the balance between pro- and anti-angiogenic factors, which leads to neovascularization. New blood and lymphatic vessels facilitate the transfer of donor antigens by mature APCs to lymph nodes in the draining eyeball. Here, donor antigens can be presented to naïve T cells (Th0), leading to the clonal expansion of T helper type 1 cells (Th1), and Th1 are mediators of graft rejection. *ANG* angiopoietin, *FGF* basic fibroblast growth factor, *PDGF* platelet-derived growth factor, *PEDF* pigment epithelium-derived factor, *sVEGFR* soluble vascular endothelial growth factor

Due to the immune-privileged location, corneal transplantation typically has better outcomes than SOT. The 10-year survival rate of low-risk corneal transplants is 85–90% (Pramanik et al. 2006; Thompson et al. 2003). Low-risk patients typically do not require systemic immunosuppression (IS) and are successfully treated with corticosteroid eye drops (Nguyen et al. 2007) (Figs. 1a and 2). In high-risk corneal transplant recipients (e.g., inflamed or consecutive transplants) (Fig. 1b, c and Fig. 3), vascularized (Fig. 1d and 4) allograft rejection occurs in 40–70% cases/year (Fig. 1e–f, Table 1). The immune response is the main cause of corneal graft failure and loss of its transparency (Kamp et al. 1995; Williams et al. 2008), which are treated by variable strategies depending on the transplant center.

**Table 1:** Characteristics of low- and high-risk keratoplasties

	Low risk	High risk	
Indications for transplantation	Keratoconus (noninflammatory ectatic corneal disease with corneal thinning and its surface distortion) Corneal dystrophies (noninflammatory, genetic corneal disorders often with accumulation of abnormal material) Corneal scars and opacities (without neovascularization)	Infectious diseases (bacterial, fungal, viral) Inflammatory diseases (use of steroids or inflammation at the moment of the surgery) Retransplantations Corneal neovascularization (due to chemical injury, previous infections)	Collaborative Corneal Transplantation Studies Research Group (1992), Maguire et al. (1994), Hahn et al. (1995), Hill (1989), Williams et al. (2008)
Procedure	Lamellar (partial-thickness) or penetrating keratoplasty	Penetrating (full-thickness) keratoplasty	
Sutures	Single or double continuous, combination of interrupted and continuous sutures	Interrupted suture	Lee et al. (2012)
Risk of graft rejection	≤ 10% cases in 5 years	40–70% cases a year (*p* < 0.05)	Kamp et al. (1995), Williams et al. (2008), Nguyen et al. (2007), Thompson et al. (2003), Pramanik et al. (2006)

In terms of immunobiology, the cornea has unique features that differentiate it from other organs. However, the immune privilege is compromised in high-risk corneal transplant recipients (Di Zazzo et al. 2020). In these patients, the effects of donor-specific anti-human leukocyte antigen (HLA) antibodies (donor-specific antibodies: DSA) on short- and long-term graft outcomes might be underestimated. Its knowledge could change the approach to the prevention and treatment of graft rejection. Since 1969, several studies have demonstrated the clinical implications of DSA monitoring in SOTs (e.g., Patel and Terasaki 1969; Terasaki et al. 1971). The presence of anti-HLA antibodies after kidney, heart, or lung transplantation is associated with worse graft survival (Campbell 2013). Currently, the detection of anti-HLA antibodies particularly those directed against the donors’ HLA is one of the most important approaches used in organ transplant recipients. The major histocompatibility complex (MHC) is highly polymorphic, which means that many different allelic products of the genes are possible, so the likelihood of finding two HLA identical organisms is very low (Klein and Sato 2000). Since HLA antigens are expressed on the surface of every nucleated cell, DSA directed against them may limit the success of transplantation.

### Immune Privilege

Ocular immune privilege is an evolutionary adaptation based on reduction of the immune response to foreign antigens to protect vulnerable structures and preserve vision (Medawar 1948); it prevents damage caused by inflammation. There are three barriers that contribute to corneal immune privilege (Table 2).

**Table 2:** Mechanisms of ocular immune privilege

Barrier	Mechanism	References
Anatomical	Lack of blood and lymphatic vessels (healthy cornea is avascular) Blood-ocular barrier (tight junctions between cells)	Cunha-Vaz et al. (2011)
Cellular	Small number of mature APCs	Hamrah et al. (2003), Hamrah and Dana (2007), Knickelbein et al. (2009)
Molecular	Constitutive expression of Fas ligand (FasL; CD 95L) inducing apoptosis of cells expressing Fas such as activated T lymphocytes Immunosuppressive cytokines modulating the host immune response: TGF-β and melanocyte-stimulating hormone Complement-regulatory cytokines maintaining low complement activity B7-H1 molecule inducing T cell apoptosis via programmed cell death protein 1 Anterior chamber-associated immune deviation downregulating antigen-specific delayed type hypersensitivity and promoting humoral response with reduction of complement-fixing antibodies	Ferguson and Griffith (2006), Taylor (2009), Sohn et al. (2000), Hori et al. (2006), Wilbanks and Streilein (1991), Skelsey et al. (2003)

These mechanisms facilitate immune tolerance to donor antigens. Moreover, the corneal epithelium, keratocytes, and endothelium do not express HLA class II antigens and the expression of HLA class I antigens on the surface of these cells is restricted (Whitsett and Stulting 1984). Interestingly, studies using mouse and rat models of corneal transplants have revealed that minor HLA-H antigens including the male-specific minor histocompatibility antigen H-Y are expressed on corneal cells, and some may terminate ocular immune privilege and initiate graft rejection (Haskova et al. 2003; Larkin et al. 1995; Streilein et al. 2003). The expression of MHC class I and class II antigens changes during inflammation, vascularization, and regrafting, when more antigen-presenting cells (APCs) are present in all corneal layers and are more prone to expressing MHC II and presenting donor antigens to lymphocytes (Hamrah et al. 2003; Hamrah and Dana 2007; Knickelbein et al. 2009).

In high-risk corneal transplant recipients, there are three main factors that abolish immune privilege: vascularization of corneal tissue, ocular inflammation, and previous graft rejection disturbing the corneal microenvironment (Fig. 3). Pre-graft corneal neovascularization is always accompanied by lymphangiogenesis, and lymph vessels are the direct path of APCs to the lymph nodes (Figs. 1d and 4). In most studies, the cornea with two or more quadrants of vascularization is defined as “high-risk”, as there is a correlation between the number of vascularized quadrants and the incidence of graft rejection and graft failure (Collaborative Corneal Transplantation Studies 1992; Williams et al. 2018). The expected 1-year graft survival in patients with non-vascularized corneas is reportedly 95%, in contrast to 78% if vascularization is present in the four quadrants of the cornea (*p* < 0.001). After 8 years of follow-up, the survival rates are 73% and 32%, respectively (*p* < 0.001) (Williams et al. 2018). Furthermore, active inflammation and/or steroid use at the time of grafting are independent risk factors for worse graft survival (Williams et al. 2018).

It is recommended that the surgery in high-risk recipients was, if possible, elective. Currently more frequently performed lamellar procedures are often not possible in these cases and full-thickness (penetrating) keratoplasties are chosen. The technique of corneal suturing does not affect the rejection rate, but it is recommended to hide the knot under the surface of the donor’s cornea because if it is in the patient's own cornea, it is more likely that irritation will stimulate vascular growth (Maguire et al. 1994; Williams et al. 2018). It is also recommended for high-risk cases to use interrupted suture technique, as there might be a greater risk of early suture loosening (and a single loose suture can be removed easily without the risk of wound dehiscence) (Lee et al. 2012).

According to the Australian corneal graft registry, a history of previous graft loss is the indication for corneal transplantation in 25% of cases. Moreover, reduced corneal graft survival and an increased risk of graft rejection is correlated with the number of retransplantations (Collaborative Corneal Transplantation Studies Research Group 1992; Williams et al. 2018).

### Mechanisms of Corneal Graft Rejection

Corneal graft rejection occurs after immune privilege is compromised (Figs. 1e, f, 3, and 4). The evidence on corneal graft rejection is mainly based on animal studies. Graft rejection is predominantly cell-mediated, and both indirect and direct pathways may play a role in allorecognition (Pleyer and Schlickeiser 2009). The significance of allospecific antibodies remains unclear, but there is evidence for donor-derived antibody involvement in the cytolysis of corneal cells.

### The Role of Antibodies in Corneal Graft Rejection

Collaborative Corneal Transplantation Studies data have shown no benefit of HLA matching in corneal transplantation, but have revealed that ABO blood group matching may improve corneal graft survival (Collaborative Corneal Transplantation Studies Research Group 1992). However, recent studies using modern methods for HLA typing have demonstrated that HLA matching may enhance corneal graft survival in high-risk patients (Bartels et al. 2003; Khaireddin et al. 2003; Völker-Dieben et al. 2000). As in the high-risk setting, the APCs of corneal donors express high levels of MHC II and costimulatory molecules and the direct pathway of allorecognition is activated, MHC matching may be useful (Hamrah et al. 2003). Studies have shown that corneal graft rejection is cell-mediated (Larkin et al. 1997; Niederkorn 2007; Sonoda et al. 1995). However, the majority of these studies used animal models, because corneal buttons in humans are usually examined months after the onset of rejection as well as after steroid treatment (Larkin et al. 1997). Finally, as animal and human tissues differ and rejection proceeds differently, there are problems extrapolating animal model data to clinical settings (George and Larkin 2004).

Some studies have indicated the possible role of circulating DSA (Hahn et al. 1995; Roy et al. 1992; Sel et al. 2012). Roy et al. (1992) showed that pretransplant panel-reactive antibodies are not associated with corneal graft rejection, but the production of antibodies after surgery (in both HLA-A and HLA-B compatible and incompatible recipients) has a negative impact on corneal graft survival and increases the risk of endothelial rejection (Roy et al. 1992). Hanh et al. (1995) showed that the presence of lymphocytotoxic antibodies (particularly directed against donor class I HLA) in patients after high-risk keratoplasties was associated with immune-mediated graft failure and thus may be indicative of corneal graft rejection. Other investigators have argued that the presence of anti-donor antibodies does not have any predictive value; for example, Jager et al. (1994) found that recipients with keratoconus and graft failure due to non-immunological mechanisms (e.g., graft decompensation) were significantly more often positive for anti-donor antibodies than healthy controls. Hargrave et al. (2003) concluded that although transplanted corneal tissue is capable of stimulating the production of allospecific antibodies directed against histocompatibility antigens, the production of IgG alloantibodies does not appear to correlate with corneal graft rejection and can occur in the absence of DSA.

While there are no routine tests for determining the cellular immune status of the recipient, sensitive methods for detecting HLA antibodies have been developed. The introduction of solid-phase immunoassays has enabled the rapid identification of both complement- and non-complement-dependent antibodies by the enzyme-linked immunosorbent assay and flow cytometry (Abbes et al. 2017). Thus, HLA antibody testing has become the gold standard for the clinical management of SOT recipients.

In the study by Sel et al. (2012), 45 low- and high-risk corneal transplant recipients had DSA analyzed before and after transplantation, and were followed up for 18 months. The authors found that 75% of patients with preformed DSA suffered from immunological complications, including complete graft loss in four cases during the first 2 months. In contrast, 77% of recipients without preformed DSA had no immunological complications during the observation time. Even though there are differences between the immunologic mechanisms of corneal transplantation and SOT, they might be less important than the similarities; thus, more attention should be paid to high-risk corneal recipients, especially corneal regrafts.

### Can Donor-Specific Anti-HLA Antibodies Contribute to Corneal Graft Rejection?

In 1969 it was demonstrated that antibodies directed against MHC antigens are the main cause of rejection in SOT (Patel and Terasaki 1969). HLA sensitization to donor antigens may occur before or after transplantation (Abbes et al. 2017; Jordan et al. 2004; Taylor et al. 2007). The risk factors for anti-HLA antibody production are previous transplantation (50–76% of patients with HLA seroconversion; *p* < 0.0001), blood transfusion, or pregnancy (Worthington et al. 2003). The adaptive immune response is antigen-specific, and the different pathways of allorecognition (direct, indirect, semidirect) have not yet been described in corneal graft rejection (Moreau et al. 2013).

For many years, the T cell response was considered predominant in SOT but it is now known that the humoral response is the main cause of acute graft loss (Terasaki 2003). The presence of preexisting DSA is correlated with antibody-mediated rejection (AMR) and kidney graft loss (Lefaucheur et al. 2010). Similarly, patients who develop de novo DSA after surgery, have worse graft outcomes (Cooper et al. 2011). Some DSA uses complement fixation as their primary mechanism for antibody-mediated SOT damage (Yell et al. 2015). In addition, the presence of C1q binding de novo DSA is an independent risk factor for AMR and kidney graft loss (Yabu et al. 2011).

There are different mechanisms underlying corneal graft rejection. Animal studies on B cell-deficient and complement-deficient mice showed that it can occur in the absence of complement-fixing antibodies (Goslings et al. 1999). Accordingly, some researchers concluded that DSA play minor or no role in corneal graft rejection. However, it is currently known that alloantibodies can also cause serious injury to the corneal button. Endothelial cells are most important in maintaining corneal transparency but are also the most vulnerable to complement-dependent and complement-independent lysis by cytotoxic antibodies in vitro (Hargrave et al. 2003). Keratocytes localized in the corneal stroma and epithelial cells are also prone to antibody-mediated destruction (Hargrave et al. 2003). Interestingly, animal studies have shown that the passive transfer of serum containing cytotoxic antibodies against graft donor antigens significantly accelerates the onset of corneal transplant rejection and shortens graft survival (Holáň et al. 2005).

In summary, allospecific antibodies are not always the cause of corneal graft decompensation but indeed can cause serious graft damage in a complement-dependent or complement-independent manner. Furthermore, there are four types of corneal graft rejection: acute or chronic, each of which can be cellular- or antibody-mediated. Of course, overlapping types are also possible. Specific approaches should be applied depending on the type of rejection.

### Impact of DSA Detection on Treatment Strategies

DSA monitoring has become a mainstay of AMR risk stratification in SOT recipients; however, it is not routinely performed after keratoplasties. Establishing a link between pre-transplant sensitization or development of antibodies de novo, and individualized decision making in this patient population requires large, prospective randomized clinical trials with the use of modern DSA detection methods. At present, it seems reasonable to stratify the immunological risk for graft rejection in cornea recipients based on pre-existing DSA. Further adjustments of IS could follow such stratification.

Systemic IS in patients after high-risk keratoplasty seems to improve graft survival but rejection episodes still occur in up to 40% of grafts after 5 years (Chow et al. 2015). Corticosteroids have been used in ophthalmology since the 1950s, and remain the cornerstone of IS therapy for corneal graft rejection prevention and treatment (Crawford et al. 2013). In the 1980’s, systemic cyclosporine A (CsA) was introduced in high-risk corneal transplant recipients. It is used to treat graft rejection rather than function as a prophylaxis (Hill 1989; Shimazaki et al. 2011). Reis et al. (1999) and Szaflik et al. (2016) confirmed that mycophenolate mofetil can be an effective alternative to CsA for the prevention of corneal graft rejection. Systemic tacrolimus is not wildly used in ophthalmology. The treatment regimens used in latter studies were based on experience in SOTs, despite the fact that corneal transplant immunobiology significantly differs from vascularized organs or bone marrow transplantations. Therefore, the question arises of whether such treatment regimens are physiologically and immunologically justified. It seems reasonable to validate DSA utility assessments, both pre-transplant as well as de novo formation after transplantation for several reasons. First, corneal graft rejection mechanisms differ from those in SOT. Second, at least four types of corneal graft rejection that may require different therapeutic approaches should be distinguished. If the role of antibodies in corneal graft rejection is confirmed clinically, their evaluation will help define diagnostic and therapeutic options for corneal transplantation. For example, patients with positive DSA at higher risk of rejection before second or third transplantations could receive personalized therapy. Such desensitization strategies based on plasma exchange, intravenous immunoglobulin infusion, anti-CD20 or anti CD38 monoclonal antibodies, proteasome inhibitors, complement inhibitors, or interleukin-6 blockers are used in SOT (Schinstock et al. 2021). Additionally, DSA may be the cause of rapid graft decompensation and the relatively poor response to treatment in many cases.

## Conclusion

The impact of DSA on corneal graft rejection still remains unknown but determining the role of pre-existing and/or de novo DSA could advance our understanding of corneal graft rejection mechanisms. This may help stratify the immunological risk of rejection and lead to personalized treatment for this group of transplant recipients.

## Data Availability

Not applicable.

## References

[CR1] Abbes S, Metjian A, Gray A (2017). Human leukocyte antigen sensitization in solid organ transplantation: a primer on terminology, testing, and clinical significance for apheresis practitioner. Ther Apher Dial.

[CR2] Bartels MC, Doxiadis II, Colen TP (2003). Long-term outcome in high-risk corneal transplantation and the influence of HLA-A and HLA-B matching. Cornea.

[CR3] Campbell P (2013). Clinical relevance of human leukocyte antigen antibodies in liver, heart, lung and intestine transplantation. Curr Opin Organ Transpl.

[CR4] Chow SP, Cook SD, Tole DM (2015). Long-term outcomes of high-risk keratoplasty in patients receiving systemic immunosuppression. Cornea.

[CR5] Collaborative Corneal Transplantation Studies Research Group (1992). The collaborative corneal transplantation studies (CCTS). Effectiveness of histocompatibility matching in high-risk corneal transplantation. The Collaborative Corneal Transplantation Studies Research Group. Arch Opthalmol.

[CR6] Cooper JE, Gralla J, Cagle L (2011). Inferior kidney allograft outcomes in patients with de novo donor-specific antibodies are due to acute rejection episodes. Transplantation.

[CR7] Crawford AZ, Patel DV, McGhee CN (2013). A brief history of corneal transplantation: from ancient to modern. Oman J Ophthalmol.

[CR8] Cunha-Vaz J, Bernardes R, Lobo C (2011). Blood-retinal barrier. Eur J Ophthalmol.

[CR9] Delbosc B, Fellmann D, Piquot X (1990). HLA antigenicity of normal and pathological corneas. J Fr Ophtalmol.

[CR10] Di Zazzo A, Lee SM, Sung J (2020). Variable responses to corneal grafts: insights from immunology and systems biology. J Clin Med.

[CR11] Ferguson TA, Griffith TS (2006). A vision of cell death: Fas ligand and immune privilege 10 years later. Immunol Rev.

[CR12] George AJ, Larkin DF (2004). Corneal transplantation: the forgotten graft. Am J Transpl.

[CR13] Goslings WR, Yamada J, Dana MR (1999). Corneal transplantation in antibody-deficient hosts. Invest Ophthalmol vis Sci.

[CR14] Hahn AB, Foulks GN, Enger C (1995). The association of lymphocytotoxic antibodies with corneal allograft rejection in high risk patients. The Collaborative Corneal Transplantation Studies Research Group. Transplantation.

[CR15] Hamrah P, Dana MR (2007). Corneal antigen-presenting cells. Chem Immunol Allergy.

[CR16] Hamrah P, Liu Y, Zhang Q (2003). Alterations in corneal stromal dendritic cell phenotype and distribution in inflammation. Arch Ophthalmol.

[CR17] Hargrave SL, Mayhew E, Hedge S (2003). Are corneal cells susceptible to antibody-medaited killing in corneal graft rejection?. Transpl Immunol.

[CR18] Haskova Z, Sproule TJ, Roopenian DC (2003). An immunodominant minor histocompatibility antigen that initiates corneal allograft rejection. Transplantation.

[CR19] Hill JC (1989). The use of cyclosporine in high-risk keratoplasty. Am J Ophthalmol.

[CR20] Holáň V, Vítová A, Krulová M (2005). Susceptibility of corneal allografts and xenografts to antibody-mediated rejection. Immunol Lett.

[CR21] Hong JW, Liu JJ, Lee JS (2001). Proinflammatory chemokine induction in keratocytes and inflammatory cell infiltration into the cornea. Invest Ophthalmol vis Sci.

[CR22] Hori J, Wang M, Miyashita M (2006). B7–H1-induced apoptosis as a mechanism of immune privilege of corneal allografts. J Immunol.

[CR23] Jager MJ, Vos A, Pasmans S (1994). Circulating cornea-specific antibodies in corneal disease and cornea transplantation. Graefes Arch Clin Exp Ophthalmol.

[CR24] Janyst M, Kaleta B, Janyst K (2020). Comparative study of immunomodulatory agents to induce human T regulatory (Treg) cells: preferential Treg-stimulatory effect of prednisolone and rapamycin. Arch Immunol Ther Exp.

[CR25] Jordan SC, Tyan D, Stablein D (2004). Evaluation of intravenous immunoglobulin as an agent to lower allosensitization and improve transplantation in highly sensitized adult patients with end-stage renal disease: report of the NIH IG02 trial. J Am Soc Nephrol.

[CR26] Kamp MT, Fink NE, Enger C (1995). Patient-reported symptoms associated with graft reactions in high-risk patients in the Collaborative Corneal Transplantation Studies. Cornea.

[CR27] Khaireddin R, Wachtlin J, Hopfenmüller W (2003). HLA-A, HLA-B and HLA-DR matching reduces the rate of corneal allograft rejection. Graefes Arch Clin Exp Ophthalmol.

[CR28] Klein J, Sato A (2000). The HLA system. First of two parts. N Engl J Med.

[CR29] Knickelbein JE, Watkins SC, McMenamin PG (2009). Stratification of antigen-presenting cells within normal cornea. Ophthalmol Eye Dis.

[CR30] Kuffová L, Holán V, Lumsden L (1999). Cell subpopulations in failed human corneal grafts. Br J Ophthalmol.

[CR31] Larkin DF, Takano T, Standfield SD (1995). Experimental orthotopic corneal xenotransplantation in the rat. Mechanisms of graft rejection. Transplantation.

[CR32] Larkin DF, Calder VL, Lightman SL (1997). Identification and characterization of cells infiltrating the graft and aqueous humor in rat corneal allograft rejection. Clin Exp Immunol.

[CR33] Lee RM, Lam FC, Georgiou T (2012). Suturing techniques and postoperative management in penetrating keratoplasty in the United Kingdom. Clin Ophthalmol.

[CR34] Lefaucheur C, Loupy A, Hill GS (2010). Preexisting donor-specific HLA antibodies predict outcome in kidney transplantation. J Am Soc Nephrol.

[CR35] Maguire MG, Stark WJ, Gottsch JD (1994). Risk factors for graft failure and rejection in collaborative corneal transplantation studies. Collaborative Corneal Transplantation Studies Research Group. Ophthalmology.

[CR36] Maier P, Heizmann U, Böhringer D (2011). Predicting the risk for corneal graft rejection by aqueous humor analysis. Mol vis.

[CR37] Medawar P (1948). Immunity of homologous grafted skin; the fate of skin homografts transplanted to the brain, to subcutaneous tissue, and to the anterior chamber of the eye. Br J Exp Pathol.

[CR38] Moreau A, Varey E, Anegon I (2013). Effector mechanisms of rejection. Cold Spring Harb Perspect Med.

[CR39] Nguyen NX, Seitz B, Martus P (2007). Long-term topical steroid treatment improves graft survival following normal-risk penetrating keratoplasty. Am J Ophthalmol.

[CR40] Niederkorn JY (2007). Immune mechanisms of corneal allograft rejection. Curr Eye Res.

[CR41] Patel R, Terasaki PI (1969). Significance of the positive crossmatch test in kidney transplantation. N Engl J Med.

[CR42] Pleyer U, Schlickeiser S (2009). The taming of the shrew? The immunology of corneal transplantation. Acta Ophthalmol.

[CR43] Pramanik S, Musch DC, Sutphin JE (2006). Extended long-term outcomes of penetrating keratoplasty for keratoconus. Ophthalmology.

[CR44] Reis A, Reinhhard T, Voiculescu A (1999). Mycophenolate mofetil versus cyclosporine A in high risk keratoplasty patients: a prospectively randomized clinical trial. Br J Ophthalmol.

[CR45] Roy R, Boisjoly HM, Wagner E (1992). Pretransplant and posttransplant antibodies in human corneal transplantation. Transplantation.

[CR46] Schinstock C, Tambur A, Stegall M (2021). Current approaches to desensitization in solid organ transplantation. Front Immunol.

[CR47] Schönberg A, Hamdorf M, Bock F (2020). Immunomodulatory strategies targeting dendritic cells to improve corneal graft survival. J Clin Med.

[CR48] Sel S, Schlaf G, Schurat O (2012). A novel ELISA-based crossmatch procedure to detect donor-specific anti-HLA antibodies responsible for corneal allograft rejections. J Immunol Methods.

[CR49] Shimazaki J, Den S, Omoto M (2011). Prospective, randomized study of the efficacy of systemic cyclosporine in high-risk corneal transplantation. Am J Ophthalmol.

[CR50] Skelsey ME, Mayhew E, Niederkorn JY (2003). Splenic B cells act as antigen presenting cells for the induction of anterior chamber-associated immune deviation. Invest Ophthalmol vis Sci.

[CR51] Sohn JH, Kaplan HJ, Suk HJ (2000). Chronic low level complement activation within the eye is controlled by intraocular complement regulatory proteins. Invest Ophthalmol vis Sci.

[CR52] Sonoda Y, Sano Y, Ksander B (1995). Characterization of cell-mediated immune responses elicited by orthotopic corneal allografts in mice. Invest Ophthalmol vis Sci.

[CR53] Streilein JW, Arancibia-Caracamo C, Osawa H (2003). The role of minor histocompatibility alloantigens in penetrating keratoplasty. Dev Ophthalmol.

[CR54] Stuart PM, Griffith TS, Usui N (1997). CD95 ligand (FasL)-induced apoptosis is necessary for corneal allograft survival. J Clin Invest.

[CR55] Szaflik JP, Major J, Izdebska J (2016). Systemic immunosuppression with mycophenolate mofetil to prevent corneal graft rejection after high-risk penetrating keratoplasty: a 2-year follow-up study. Graefes Arch Clin Exp Ophthalmol.

[CR56] Taylor AW (2009). Ocular immune privilege. Eye.

[CR57] Taylor DO, Edwards LB, Boucek MM (2007). Registry of the International Society for Heart and Lung Transplantation: twenty-fourth official adult heart transplant report-2007. J Heart Lung Transpl.

[CR58] Terasaki PI (2003). Humoral theory of transplantation. Am J Transplant.

[CR59] Terasaki PI, Kreisler M, Mickey RM (1971). Presensitization and kidney transplant failures. Postgrad Med J.

[CR60] Thompson RW, Price MO, Bowers PJ (2003). Long-term graft survival after penetrating keratoplasty. Ophthalmology.

[CR61] Völker-Dieben HJ, Claas FH, Schreuder GM (2000). Beneficial effect of HLA-DR matching on the survival of corneal allografts. Transplantation.

[CR62] Whitsett CF, Stulting RD (1984). The distribution of HLA antigens on human corneal tissue. Invest Ophthalmol vis Sci.

[CR63] Wilbanks GA, Streilein JW (1991). Studies on the induction of anterior chamber-associated immune deviation (ACAID). 1. Evidence that an antigen-specific, ACAID-inducing, cell-associated signal exists in the peripheral blood. J Immunol.

[CR64] Williams KA, Lowe M, Bartlett C (2008). Risk factors for human corneal graft failure within the Australian corneal graft registry. Transplantation.

[CR65] Williams KE, Keane MC, Coffey NE et al (2018) The Australian corneal graft registry, 2018 Report. Bedford Park SA

[CR66] Worthington JE, Martin S, Al-Husseini DM (2003). Posttransplantation production of donor HLA-specific antibodies as a predictor of renal transplant outcome. Transplantation.

[CR67] Yabu JM, Higgins JP, Chen G (2011). C1q-fixing human leukocyte antigen antibodies are specific for predicting transplant glomerulopathy and late graft failure after kidney transplantation. Transplantation.

[CR68] Yell M, Muth BL, Kaufman DB (2015). C1q binding activity of de novo donor-specific HLA antibodies in renal transplant recipients with and without antibody-mediated rejection. Transplantation.

[CR69] Yoon CH, Choi SH, Lee HJ (2019). Predictive biomarkers for graft rejection in pig-to-non-human primate corneal xenotransplantation. Xenotransplantation.

[CR70] Zhu SN, Dana MR (1999). Expression of cell adhesion molecules on limbal and neovascular endothelium in corneal inflammatory neovascularization. Invest Ophthalmol vis Sci.

[CR71] Zhu S, Dekaris I, Duncker G (1999). Early expression of proinflammatory cytokines interleukin-1 and tumor necrosis factor-alpha after corneal transplantation. J Interferon Cytokine Res.

